# Physiological and Transcriptional Responses in Weaned Piglets Fed Diets with Varying Phosphorus and Calcium Levels

**DOI:** 10.3390/nu11020436

**Published:** 2019-02-20

**Authors:** Christian Gerlinger, Michael Oster, Luisa Borgelt, Henry Reyer, Eduard Muráni, Siriluck Ponsuksili, Christian Polley, Brigitte Vollmar, Martin Reichel, Petra Wolf, Klaus Wimmers

**Affiliations:** 1 Leibniz Institute for Farm Animal Biology, Wilhelm-Stahl-Allee 2, 18196 Dummerstorf, Germany; gerlinger@fbn-dummerstorf.de (C.G.); oster@fbn-dummerstorf.de (M.O.); reyer@fbn-dummerstorf.de (H.R.); murani@fbn-dummerstorf.de (E.M.); ponsuksili@fbn-dummerstorf.de (S.P.); 2 Nutrition Physiology and Animal Nutrition, University of Rostock, Justus-von-Liebig-Weg 6b, 18059 Rostock, Germany; Luisaborgelt@gmx.de (L.B.); petra.wolf@uni-rostock.de (P.W.); 3 Institute for Experimental Surgery, University Medicine Rostock, Schillingallee 69a, 18057 Rostock, Germany; christian.polley@uni-rostock.de (C.P.); brigitte.vollmar@med.uni-rostock.de (B.V.); 4 Mechanical Engineering Design/Lightweight Design, University of Rostock, Albert-Einstein-Straße 2, 18059 Rostock, Germany; martin.reichel@uni-rostock.de; 5 Animal Breeding and Genetics, University of Rostock, Justus-von-Liebig-Weg 7, 18059 Rostock, Germany

**Keywords:** calcium homeostasis, dietary mineral intake, immunomodulation, phosphorus homeostasis, trabecular bone

## Abstract

Phosphorus (P) is an important element of various metabolic and signalling processes, including bone metabolism and immune function. To elucidate the routes of P homeostasis and utilization, a five-week feeding study was conducted with weaned piglets receiving a diet with recommended amounts of P and Ca (M), or a diet with lower (L) or higher (H) P values and a constant Ca:P ratio. Routes of P utilization were deduced via bone characteristics (MicroCT), genome-wide transcriptomic profiles of peripheral blood mononuclear cells (PBMCs), and serum mineral levels. MicroCT revealed significantly lower bone mineral density, trabecular number, and mechanical fracture load in (L). Gene expression analyses showed transcripts of 276 and 115 annotated genes with higher or lower abundance in (H) than (L) that were related to basic cellular and metabolic processes as well as response to stimuli, developmental processes and immune system processes. This study shows the many molecular routes involved in P homeostasis that should be considered to improve endogenous mechanisms of P utilization.

## 1. Introduction

Phosphorus (P) and calcium (Ca) are essential elements for all living organisms. They play a vital role in growth processes, formation and stability of bones, and maintenance of a physiological cell metabolism. The homeostasis of P and Ca is largely maintained by a trifecta of absorption in the intestines, secretion/resorption in the kidneys and mobilization/storage in bones. Hormones like vitamin D (calcitriol), parathyroid hormone (PTH), fibroblast growth factor 23 (FGF23), and calcitonin as well as corresponding mineral transporters, receptors, and transcription factors help maintain Ca and P mineral homeostasis [1]. For livestock, the need for P and Ca is primarily dependent on bone formation requirements that are strongly associated with the health and welfare of the animals. In fact, favorable growth and performance traits need to be combined with adequate bone formation to ensure a well-functioning musculoskeletal system [2].

Both dietary P and Ca deficiencies and surpluses have considerable effects on bone mineral content and mineral density [3], bone composition [4], and bone development [5]. Systemic effects caused by an altered P and Ca supply indicate highly dynamic influxes and effluxes of P, Ca, and superordinate endocrine metabolites in the blood [1]. Because P and Ca-dependent processes rely on the uptake, excretion, and availability of these elements [6], the regulation of plasma PTH, calcitriol (1,25(OH)_2_D_3_), calcium-binding protein (CaBP) [7,8], and IGF-1 concentration [9] have received considerable attention. However, studies investigating adaptation to P and Ca deficiencies and surpluses in the context of adequate bone mineralization and mineral utilization are scarce.

During the growth and development of piglets, there is a central role for P and Ca in osteogenesis but also a significant demand for these minerals and their associated endocrine factors in soft tissue development [10]. In this context, vitamin D deficiency has been associated with smaller muscle fibers and altered expression of genes relevant to myogenesis [11].

Recent findings in pigs also provide increasing evidence for links between dietary P and the immune system [12]. Specifically, there are aspects of osteoimmunology that might also depend on the dietary supply of minerals [13]. Here, the two-way interaction between bone tissue and immunocompetent cells in blood could provide a significant contribution [14]. Bone and immune system development are very strongly linked by regulatory processes in the bone marrow that control immune functions and hematopoiesis, especially by signals between osteoclasts, which are monocytes derived from hematopoietic precursors, and immune cells [13]. In this context, peripheral blood mononuclear cells (PBMCs) are not only a suitable surrogate cell population for various other tissue [15], but also enable detailed phenotyping of osteoimmunological effects. Mutual research on bone and immune cells can therefore shed light on the consequences of P and Ca imbalances in primarily affected organs and tissues [16].

This study aims to identify the effects of dietary P and Ca imbalances on growth performance, bone characteristics, serum mineral levels, and expression data from PBMCs.

## 2. Materials and Methods

This study was approved by the Scientific Committee of the Leibniz Institute for Farm Animal Biology (FBN) and licensed by the Ethics Committee of the federal state of Mecklenburg-Western Pomerania, Germany (Landesamt für Landwirtschaft, Lebensmittelsicherheit und Fischerei; LALLF M-V7221.3-1-053-15).

### 2.1. Animals, Diet, and Sample Collection

As previously described [17], German Landrace piglets (*Sus scrofa domesticus*, *n* = 21) were fed three different wheat/barley/soybean-based diets varying in Ca and P levels for five weeks (days 28 to 64 of life) (Table S1). The animals were obtained from four litters sired by individual boars and were kept individually on a flat-deck. Each group comprised at least three females and three castrated males. Piglets received a medium diet, considered the control diet, with recommended mineral levels (M diet; P: 0.84%; Ca: 1.27% of dry matter) [18]; a low mineral diet (L diet; P: 0.56%; Ca: 0.79% of dry matter); or a high-mineral diet (H diet; P: 1.02%; Ca: 1.69% of dry matter; Table S1). The crude nutrients in the feed were analyzed according to the standard methods for chemical analysis of feed [19]. The resulting dietary Ca:P ratios ranged between 1.41:1 and 1.65:1. Neither microbial phytase nor other phosphatases were added. Pigs had free access to water and feed (*ad libitum*). Zootechnical parameters such as body weight (BW), daily weight gain (DWG), and daily feed intake (DFI) were recorded and the mean presented for each week (days 28, 35, 42, 49, 56, 63). Blood samples were taken on day 63 from the Vena cava cranialis. Serum was prepared and stored at −80 °C. PBMCs were isolated from 5 mL of EDTA-supplemented blood by centrifugation on a Histopaque-1077 density gradient (Sigma-Aldrich, Taufkirchen, Germany) and stored at −80 °C until further analyses. On day 64, animals were anaesthetized by electrical stunning and killed by exsanguination at the Institute’s experimental slaughter facility. The left femurs were excised and stored at −20 °C.

### 2.2. Measurement of Bone Characteristics

**Figure 1:**
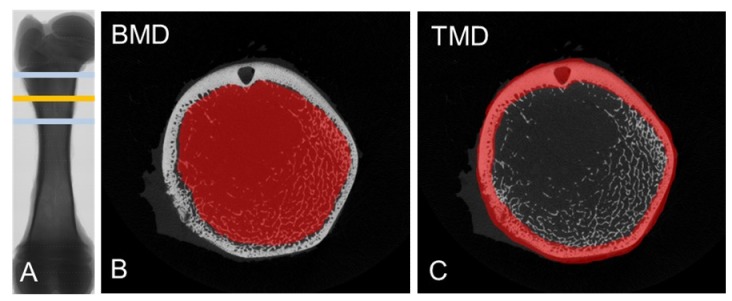
Representative images obtained from micro-CT analyses of porcine femurs. (**A**). The volume of interest (VOI) was set distal to the hip joint at 30% of the total femur length (yellow line) and covered 800 slices (light blue lines). (**B**). Region of interest (ROI) (red) represents the trabecular bone for measurements of bone mineral density (BMD). (**C**). ROI (red) to approximate the tissue mineral density (TMD) in cortical bone.

For micro-CT analyses of femurs, a high-resolution Micro-CT Imaging System was used (SkyScan 1076, Bruker-MICROCT, Kontich, Belgium). In brief, samples were thawed at 4°C in 0.9% saline for 24 h. Scans were performed with an aluminum-filter (1.0 mm), X-Ray source: 70 kV/141 µA, averaging frame: 4, rotation step: 0.6°, and pixel size: 18 µm. For sample reconstruction, a beam hardening of 30% and defect pixel masking of 20% with individual misalignment compensation and fixed histogram thresholds were used. The volume of interest (VOI) was chosen distal to the hip joint and to account for individual femur length. It was set proportionally at 30% of the total femur length (Figure 1A). The VOI covered 400 slices in both directions (800 slices in total). Cortical tissue mineral density (TMD) and trabecular bone mineral density (BMD) were measured, and a three-dimensional analysis was performed (Figure 1B,C). Microstructural parameters, including the trabecular bone volume/tissue volume ratio (BV/TV), structure model index (SMI), trabecular number (TbN), trabecular separation (TbSp), and trabecular thickness (TbTh) were obtained [20]. Fracture load and maximum deflection were measured via a three-point bending test using a servohydraulic testing machine (#858, MTS, Berlin, Germany). Specifically, the femurs were placed on their metaphysis and a displacement of 3 mm/min was applied. The data were recorded 20 times per second. The femoral diameters were recorded (min, max).

### 2.3. Measurements of Serum Parameters

Serum minerals (inorganic P, Ca) were analyzed with commercial assays using Fuji DriChem 4000i (FujiFilm, Minato, Japan). Zinc (Zn) and copper (Cu) levels were measured directly in blood serum by inductively coupled plasma optical emission spectrometry (ICP-OES) according to BVL F 0096:2013-04 [21].

### 2.4. RNA Isolation

Total RNA was isolated from PBMCs by phenol/chloroform/isopropanol extraction using the Qiazol reagent per the manufacturer’s directions (Qiagen, Hilden, Germany). Samples were treated with DNAse I (Roche, Mannheim, Germany) and purified with the column-based NucleoSpin RNA II Kit according to the manufacturer’s specifications (Macherey-Nagel, Düren, Germany). Furthermore, a NanoDrop ND-2000 spectrometer (Thermo Fisher Scientific, Schwerte, Germany) was used to quantify RNA concentrations. The samples were tested for genomic DNA contamination via PCR with primers for GAPDH (forward: AGATCCACAACCGACACGTT, reverse: CCAGAACATCATCCCTGCTT). No amplification of specific DNA targets was observed. RNA samples were stored at −80 °C until analysis and cDNA synthesis were performed.

### 2.5. Microarray Processing

For genome-wide analyses of the pig transcriptome, single stranded cDNA was synthesized, biotin-labelled, and fragmented using the WT Plus Expression kit according to the manufacturer’s instructions (Affymetrix, Santa Clara, CA, USA) [22]. The samples were hybridized on snowball arrays comprising 47,845 probe-sets [22]. The arrays were processed following the manufacturer’s instructions using the GeneChip Hybridization, Wash and Stain Kit (Affymetrix, Santa Clara, CA, USA). Raw data were generated with Affymetrix GCOS 1.1.1 software (Affymetrix, Santa Clara, CA, USA). Data were deposited in a MIAME-compliant database, the National Center for Biotechnology Information Gene Expression Omnibus (www.ncbi.nlm.nih.gov/geo; accession number: GSE122144) [23].

### 2.6. Data Analysis

Measurements on zootechnical traits, serum, and bone parameters were subjected to variance analysis (SAS Institute, Cary, NC, USA). Fixed effects represented by the dietary group, litter, and sex were considered. Tukey’s post-hoc test was applied to deduce differences between the three dietary groups. The level of significance was set at *p* < 0.05.

Expression data were processed by Expression Console (Affymetrix, version 1.4, Santa Clara, CA, USA) and R software (v3.2.3) (R Foundation for Statistical Computing, Vienna, Austria). The microarray data were subjected to quality assessment [24]. Quality control criteria were met by 20 samples; one microarray was excluded. A normalization of raw intensity data was performed by the Robust Multichip Average (RMA) approach (Log2). Probe sets with low-intensity signals were excluded from further analyses to improve statistical power [25]. The removed probe sets consisted of probe subsets that were declared present in less than 80% of all arrays. In line with phenotypic data measurements, differential expression was established by variance analysis (SAS Institute, Cary, NC, USA). To correct for multiple testing in large datasets, q-values were calculated considering the *p*-value distribution [26]. The cut-off for *q*-values was set to *q* < 0.21, which is equivalent to *p* < 0.01. To reveal differences in mRNA abundances, fold changes (FC) were calculated between dietary groups.

A functional classification analysis for molecular functions and biological processes was performed for differentially expressed transcripts obtained from the comparison of animals in the L and the H group by using the PANTHER classification system tool 13.1 (Gene Ontology, http://pantherdb.org). Additionally, the lists of differentially abundant transcripts were evaluated via Ingenuity Pathway Analysis to visualize canonical pathways and bio-functions (IPA; Ingenuity Systems, Redwood City, CA, USA). The significance of the association between pathway and data was set at *p* < 0.05 (corrected by Benjamini-Hochberg). Pathways that explicitly refer to human diseases were excluded from the results. The specified *z*-scores represent a statistical measure of the correspondence between literature knowledge of gene regulations (IPA) and the observed probe set abundance. The *z*-scores indicate an activation (positive) or inhibition (negative) of the biological functions. Absolute *z*-scores above 2 were considered relevant.

## 3. Results

This study investigated pigs fed wheat/barley-based diets with variable P and Ca contents to approximate effects on growth, bone development, and mineral-responsive molecular pathways in peripheral blood cells. Both limited and excess mineral intake prompted phenotypical and transcriptional consequences.

### 3.1. Performance and Feed Intake

**Figure 2:**
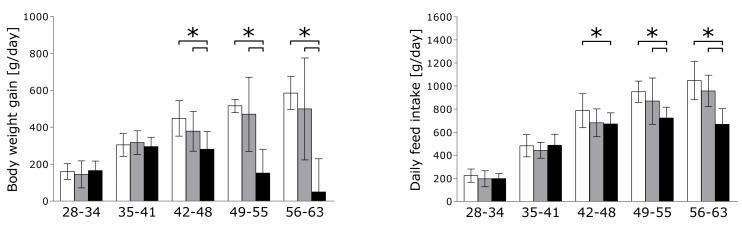
Daily weight gain and feed intake of piglets fed variable dietary amounts of P and Ca. Data refer to the weekly means of body weight gain and feed intake throughout the feeding trial (details are displayed in Table S2). Low P diet (white bars); Medium (recommended) P diet (gray bars); High P diet (black bars); * *p* < 0.05.

On day 56 and day 63, animals fed high levels of dietary P and Ca had significantly lower live weights compared to animals fed L and M (Table S2). Moreover, BWG and DFI were significantly decreased in the H group during weeks 3 (day 42–48), 4 (day 49–55), and 5 (day 56–63) (Figure 2). After 3 weeks on trial, the H-fed piglets showed clinical symptoms of parakeratosis. In some cases, the dermis showed lymphocytic infiltrates and nuclei remaining in the stratum corneum. Animals fed on L and M diets did not show any dermal irritations. Looking at the intake of Ca and P in relation to the respective weight of the animals, there is a significant difference between groups H and L over the entire course of the experiment (Table S3). In fact, animals of the H group had an absolute higher intake of P during week 1–4, while in week 5 it was not significant different from the other groups. The net P intake (g intake/day and kg BW) was higher along the whole experimental period. There was no evidence of dietary effects on performance traits of animals of the L group compared to animals of the M group.

### 3.2. Bone Characteristics

The measurements of femoral characteristics resulting from variable dietary mineral intake are given in Table 1. Animals of the L group showed significantly lower BMD and BV/TV but increased TbSp when compared to animals of the M- or H-fed groups. The TbN was lower in L compared to M animals. SMI, TMD, and TbTh were unaffected by diet. A lowered fracture load and a higher maximum deflection were observed in L animals compared to M and H animals. No statistically significant differences were observed between the animals of the M- and H-fed groups. No significant differences in femur diameters were observed between the dietary groups.

### 3.3. Serum Mineral Levels

**Figure 3:**
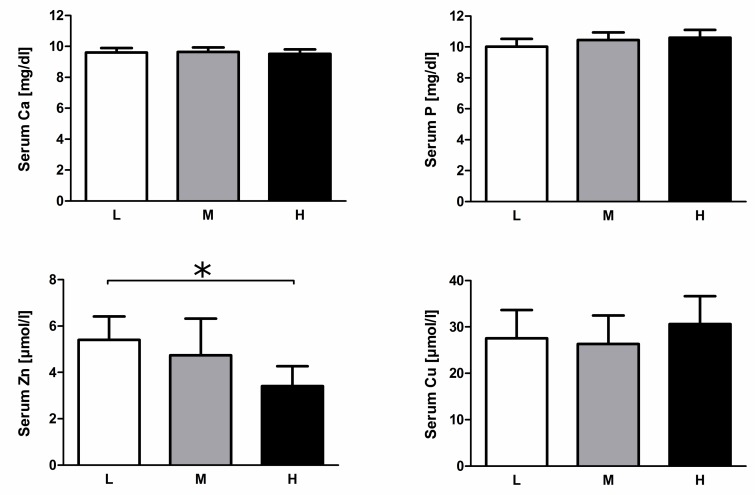
Serum inorganic P, Ca, Zn, and Cu levels of pigs fed variable dietary amounts of P and Ca. Values for serum inorganic P and Ca were obtained previously [17]. L—Low P diet; M—Medium (recommended) P diet; H—High P diet; * *p* < 0.05.

In our prior study, no significant diet-dependent differences were measured in serum levels of inorganic P and Ca [17]. Serum Zn levels were significantly decreased in animals of the H group, whereas serum Cu levels were unaffected by the dietary regimen (Figure 3).

### 3.4. PBMC Gene Expression Pattern

To deduce diet-specific mRNA expression patterns, transcriptomic profiles of PBMC were evaluated. The comparison of microarray data between the animals of the L- and the H-fed groups revealed 694 differentially expressed probe sets that passed the threshold criteria (*p* < 0.01; *q* < 0.21). These probe sets represented 391 annotated genes (276 L < H; 115 L > H) involved in 33 pathways found to be differentially expressed in L and H groups (Table 2, Table S4). To indicate differences in mRNA abundances, positive FC (L < H) and negative FC (L > H) are displayed (Table 2 and Table 3). Due to multiple testing corrections, no significant differences in gene expression were found when compared L to M or M to H groups. Therefore, these comparisons were not considered in the downstream pathway analyses.

**Figure 4:**
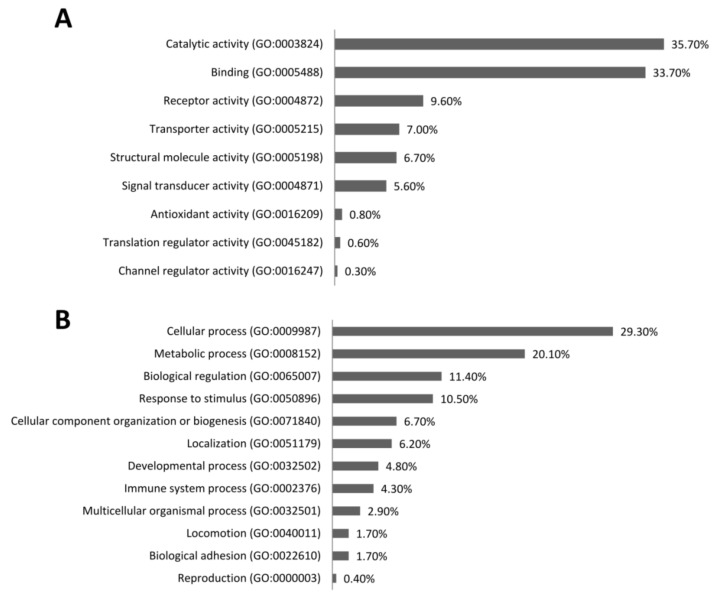
Gene Ontology (GO)-terms. Results refer to genes differentially expressed in animals fed the L and H diets. The analysis shows significantly enriched terms for (**A**) molecular functions and (**B**) biological processes.

The most abundant molecular functions (GO-terms) obtained from the overall analysis of differentially abundant transcripts (Figure 4A) in the L and H groups were catalytic activity (35.7%) and binding (33.7%). Moreover, activities of receptors (9.6%), transporters (7%), structural molecules (6.7%), and signal transducers (5.6%) were shown to be enriched. For biological processes (Figure 4B), transcripts involved in basic cellular (29.3%) and metabolic processes (20.1%) predominated. Additionally, GO-terms related to response to stimulus (10.5%), developmental processes (4.8%), and immune system processes (4.3%) were enriched.

Comparison of L and H data revealed that pathways involved in the modulation of humoral and cellular immunity were affected, i.e., “IL-10-signaling”, “IL-6 signaling”, “acute phase response signaling”, “NF-κB signaling” and “role of pattern recognition receptors in recognition of bacteria and viruses”. Moreover, analyses revealed molecular patterns that are involved in the differentiation and functions of T cells, such as “T helper cell differentiation”, the “Th1 and Th2 activation pathway”, the “Th1 pathway”, and the “Th2 pathway”. Furthermore, mRNA expression patterns associated with genes involved in monocyte and macrophage function (“Fcγ receptor mediated phagocytosis in macrophages”, “monocytes granulocyte adhesion and diapedesis”). Besides the prominence of immune pathways, genes exhibiting altered mRNA abundances were associated with pathways related to signal transduction, biosynthesis, P metabolism and gene expression machinery (“LXR/RXR activation”, “triacylglycerol biosynthesis”, “CDP-diacylglycerol biosynthesis I”, “phosphatidylglycerol biosynthesis II”, “3-phosphoinositide biosynthesis”, “d-myo-inositol (1,4,5,6)-tetrakisphosphate biosynthesis”, “d-myo-inositol (3,4,5,6)-tetrakisphosphate biosynthesis”, “superpathway of inositol phosphate compounds”, and “EIF2 signaling”). Moreover, “p38 MAPK signaling”, “PPAR signaling”, “TREM1 signaling”, “interferon signaling”, “RhoA signaling”, and “iNOS signaling” differed in L and H animals. However, the prominence of immune-related pathways is moderated by the fact that only 4.3% of enriched GO-terms mapped to “immune system process” (Figure 4B).

Table 3 displays genes showing the greatest significant differences in mRNA abundances between L and H. Here, transcripts such as C3AR1 (Complement C3a Receptor 1) and PLPP3 (phospholipid phosphatase 3) are of note known to be involved in P/Ca homoeostasis.

## 4. Discussion

A sufficient supply of minerals such as P and Ca is essential for animal health and welfare. This study examined the effects of diets with variable P and Ca levels but constant Ca:P ratio on transcriptomic and phenotypic variation in growing pigs. Furthermore, this study emphasizes the interactions of bone tissue and immunocompetent cells.

The two most striking diet-dependent differences in this study were the significant reductions of BWG and DFI in the H group starting around day 42 of life. This is consistent with other studies indicating there is an optimal quantity of dietary P and Ca content for growth [27,28]. Since P is necessary for muscle growth and bone formation, pigs preferentially develop muscle and bone during the first growth phase instead of forming fat [29]. The reduced DFI resulted in decreased weight gain and subsequently a reduced protein intake in the H-group animals on day 49 and day 63. An impact on bone growth by reducing the availability of proteins prior to bone mineralization is conceivable, as has been shown in humans challenged with protein deficiency [30]. However, only long-term impairment was observed in pigs after protein deficiency [31]. Accordingly, no differences in the cortical structure (TMD, Table 1) or femur length [17] were observed in this study. Nevertheless, findings on the difference in DFI caused by the amount of available P in the diet are controversial. In studies on piglets weighing 15 to 30 kg, a difference in DFI was confirmed [27,32]. There seems to be an optimum range of P content that should not be undercut or exceeded to reach a maximum DFI [27]. Yet, there is also no observed connection between P content and DFI [33,34]. It is conceivable that the DFI in H-group animals is lowered due to a decrease in the palatability of their diet [35]. However, the diets used in this study did not have a significant influence on Ca and P serum levels [17]. Indeed, due to the release of Ca from bone storage mediated by PTH and a regulation of Ca resorption mediated by calcitriol, the serum Ca level is only subject to minor fluctuations. 

Despite the fact that the performance characteristics of H-group animals differed significantly from those of the other groups, endogenous regulatory processes controlling mineral homeostasis likely prevented impairment in TMD, SMI, and TbTh in these animals. However, femurs from L group animals had significantly lower BMD, BV/TV, and fracture load but increased TbSp and maximum deflection compared to these parameters in H fed animals. In accordance with these results, it was found that a Ca deficit reduces the BV/TV ratio but does not influence the TbTh of 32-day-old piglets [8]. Liesegang et al., show that a reduction in the dietary P level has a negative effect on BMD [3]. In this context, BW and BMD appear as independent traits, as the H and M groups differ in growth performance but not in their respective bone characteristics. This observation is consistent with a previous report where the trabecular architecture was not directly related to the weight of the animals [2]. Interestingly, diets with variable mineral intake and constant (this study) or variable [6] Ca:P ratio altered structures primarily in the spongiosa but not in the corticalis. In fact, the dietary responsiveness of the spongiosa was demonstrated in an earlier study examining dietary Ca or P restriction [3,36]. It is unclear whether these effects result from decreased bone formation or increased mineral resorption from bones. For all three experimental dietary groups, adaptive endocrine responses were observed at the level of calcitriol (decreasing levels seen with a shift from low to high Ca and digestible P diets) and PTH (increasing levels from low to high Ca and digestible P diets) [17].

A high intake of dietary P leads to more secretion of FGF23, which in turn reduces calcitriol levels [37]. Excess FGF23 leads to reduced bone mineralization, a possible explanation for the differences in the bone parameter measurements seen previously for an H group [38]. The mechanism by which P influences bone mineralization via FGF23 is unknown [37]. For bone formation, sufficient P is required to prevent a growth delay during the growth phase or the inadequate formation of hydroxyapatite [37]. In H-group pigs, bone formation by osteoblasts could be increased or bone resorption by osteoclasts could be reduced. At the very least, the balance of formation and resorption differs in the H and L groups.

The PBMC fraction represents a diverse cell population including monocytes, NK cells, and dendritic cells that are part of the innate immune system as well as components of the adaptive immune system (B- and T-lymphocytes) [39]. Osteoclasts are monocyte-derived cells and cell types from the fraction of PBMCs are potentially involved in the regulation of bone turnover; therefore, it is meaningful to measure the expression of genes in PBMCs, which can serve as a surrogate tissue [15,40,41]. The five transcripts with the highest positive FC (IL22RA2, RNF128, ARG2, C3AR1, IL1R2) derived from comparison of the PBMC expression pattern between animals in the L- and H-fed groups are all involved in immune response regulation, including inflammation and T-helper cell function, and in signaling processes [42,43]. Their expression patterns might modify the balance between bone formation and bone resorption. Specifically, T-cells are thought to be involved in bone remodeling processes [41]. In this context, the expression of C3AR in activated T-lymphocytes links the complement system with the adaptive immune system in a manner dependent on T-lymphocytes [44]. The influence of C3AR1 on bone cell performance may indicate a modification of bone remodeling favoring osteoclast formation and the inflammatory response of osteoblasts directed by calcitriol and by involvement of the complement system and IL1β [45]. In addition, IL1R2 might affect osteoclastogenesis via the putative effects of IL1 on the expression of M-CSF by marrow stromal cells [46]. Perhaps this decoy receptor (IL1R2) acts via some mechanism to counteract excessive weakening of bone structure and for the parallel maintenance of P and Ca homeostasis. Also, three of the five transcripts with the highest negative FC (SPTSSA, PLPP3, EEF1A1) showed involvement in the regulation of inflammation and T-cell function [47,48,49,50,51]. Their involvement in inflammatory processes and the modification of Th1-specific expression by these genes could influence bone remodeling. Downregulation of pro-osteoblastic activity via the Wnt pathway by PLPP3 potentially leads to reduced bone formation and supports the results of reduced bone parameters in the L group [49]. 

Transcripts differentially expressed in the H and L groups were assigned to gene ontology terms, primarily cellular and metabolic processes, with only 4.3% being related to immune system processes. However, GO-terms like “response to stimulus” and “immune system process” indicate an active immune response contributes to the observed transcriptional differences. Pathways involved in the modulation of the humoral and cellular immunity and the differentiation and functions of T-cells were affected by dietary treatment. 

Serum Zn levels were significantly lower only in animals of the H group, indicating interactions between low dietary Zn and high Ca supplementation. In fact, the ratio of Ca and Zn in the feed and the formation of complexes of dietary phytate with Zn and Ca can ultimately reduce net Zn absorption and increase Zn requirements [52,53]. Though dietary Zn levels of 36.9–39.1 mg/kg DM (Table S1) meet standard requirements, interactions between dietary Ca and Zn led to reduced Zn serum levels [54]. The reduced DFI leads to a reduced Zn intake in the H group in week 5 only. However, reduced DFI did not result in different net Zn intake (g intake/day and kg BW) between the groups L and H (Table S3). Indeed, Zn levels modulate different aspects of the immune system. Consequently, the combination of moderate dietary Zn and high dietary Ca might contribute to the observed shifts in immune pathways and ultimately affect health and cause parakeratosis [55].

The induction of the NF-κB pathway is a key sign of inflammation leading to further proinflammatory processes like cytokine expression, which is a basic requirement for T helper cell differentiation and activation [56]. The mitogenesis of T cells, which induce the expression of VDR after activation, is inhibited by calcitriol via the MAPK pathway [57]. This indicates a possible connection between the increased immune response and lower calcitriol level in the H group [17]. 

Other functional groups influenced by differentially abundant genes were phospholipid synthesis and signaling pathways. The involvement of the “Superpathway of Inositol Phosphate Compounds” could indicate regulation of P metabolism is related to phospholipid synthesis and cell proliferation [58,59]. The “TREM1 Signaling” pathway leads to a proinflammatory response and the activation of the “p38 MAPK Signaling” pathway, which is connected to the “Interferon Signaling” pathway in immune responses [60,61]. The activation of TREM1 is triggered by NF-κB and leads to the production of proinflammatory cytokines [62]. Immune response and differentiation as well as transcription and translation are regulated in part by the “Interferon Signaling”-pathway [60]. The “iNOS signaling” pathway and the induction of NO production have several effects. From an immunological perspective, these phenomena play a cytotoxic role in the nonspecific immune response against pathogens and facilitate the influx of inflammatory cells into tissues [62]. Furthermore, NO regulates the immune response by inhibiting T and B cell proliferation and influencing T cell responses, the up- and downregulation of cytokines/chemokines/growth factors, and the modulation of signal cascades (MAPK) or transcription factors (NF-κB) [62]. Regarding nutrient homeostasis, the interaction of NO with metallothionein leads to a release of Zn [63]. Other signaling pathways like “EIF2 Signaling”, “PPAR signaling” or “RhoA signaling” are involved in the initiation or regulation of transcription or mRNA translation, thus participating in protein biosynthesis [64,65,66,67]. PPAR is interconnected with the functions of RXR and LXR in lipid metabolism. The PPAR/RXR heterodimer regulates the degradation of lipids and LXR regulates lipid synthesis [66]. “EIF2 Signaling”, “PPAR Signaling” and “LXR/RXR Activation” had negative *z*-scores, indicating inactivation of these pathways in the H group. This could reflect secondary effects due to the lower growth rate of animals in the H group.

## 5. Conclusions

Compared to animals on a medium or low Ca and P diet, piglets on a high Ca and P diet exhibited reductions in growth performance and health status. However, a ~30% reduction in dietary P levels led to consecutive adaptations in bone. Reduced P uptake had an effect on the trabecular tissue as indicated by a higher TbSp and lower TbN, likely influencing bone stability. An increase in the dietary P content had no positive effect on bones or overall pig performance. In fact, transcriptional changes in PBMC indicate a possible negative effect of high dietary P. A comparison of gene expression in the L and H groups revealed very prominent changes in transcription, many related to immune response and signalling processes involved in growth. A more detailed temporal-spatial dissection of the effects of increased dietary P and Ca contents under varying dietary mineral supplies is warranted.

## Figures and Tables

**Table 1:** Densiometric and morphometric characteristics of femurs excised from 64-day-old piglets fed variable dietary amounts of P and Ca.

		L		M		H	
Item	Unit	Mean	SD	Mean	SD	Mean	SD
Tissue mineral density (TMD)	g/mm^3^	1.06	0.03	1.07	0.04	1.10	0.02
Bone mineral density (BMD)	g/mm^3^	0.08 ^a^	0.02	0.16 ^b^	0.03	0.15 ^b^	0.04
Bone volume/Total volume (BV/TV)	%	16.68 ^a^	3.69	25.31 ^b^	6.05	23.80 ^b^	5.22
Structural model index (SMI)		1.09	0.19	1.20	0.73	1.23	0.49
Trabecular number (TbN)	1/mm	0.94 ^a^	0.19	1.39 ^b^	0.21	1.23 ^a,b^	0.49
Trabecular separation (TbSp)	mm	3.44 ^b^	1.87	0.95 ^a^	0.51	1.13 ^a^	1.03
Trabecular thickness (TbTh)	mm	0.18	0.01	0.18	0.02	0.31	0.33
Fracture load	N	764.9 ^a^	110.9	999.9 ^b^	153.2	920.4 ^b^	208.2
Maximum deflection	mm	6.5 ^b^	2.0	4.6 ^a^	1.1	4.1 ^a^	0.7

^a,b^ Indicate significant differences between groups (*p* < 0.05). L—Low P diet; M—Medium P diet; H—High P diet; SD—Standard Deviation.

**Table 2:** Excerpt of pathways altered in PBMC of piglets fed diets with low and high amounts of P and Ca. Pathways were selected for affiliation with immune response, biosynthesis, and signaling. A full list of pathways is displayed in Table S4.

Pathway Name	*z*-Score	Responsive Transcripts
**Biosynthesis**		
Superpathway of Inositol Phosphate Compounds *	-	*ACP1, DUSP2, IPMK, PIK3C2A, PIP5K1B, PPP1R12A, PTEN, PTPN12, SET, SOCS3, TNS3*
EIF2 Signaling *	1.13	*ATF3, EIF3K, PIK3C2A, RPL12, RPL7, RPL24, RPL34, RPS8, RPS19, RPS26, RPSA, SOS2*
CDP-diacylglycerol Biosynthesis I †	-	*CDS1, GPAT3, GPAT4, LPCAT2, MBOAT7*
Triacylglycerol Biosynthesis †	-	*DGAT2, GPAT3, GPAT4, LPCAT2, MBOAT7, PLPP3*
Interferon Signaling *	2.00	*IFIT3, IFNGR1, JAK2, OAS1*
**Immune Response**		
IL-10 Signaling ‡	-	*ARG2, CCR1, HMOX1, IL1A, IL1R2, IL1RAP, IL4R, IL18, IL18RAP, SOCS3*
IL-6 Signaling ‡	2.89	*CSNK2A1, IL1A, IL1R2, IL1RAP, IL6R, IL18, IL18RAP, JAK2, PIK3C2A, SOCS3, SOS2, TNFRSF1A*
Acute Phase Response Signaling ‡	2.11	*C4BPA, F8, HMOX1, IL1A, IL1RAP, IL6R, IL18, JAK2, RIPK1, SOCS3, SOD2, SOS2, TNFRSF1A*
NF-κB Signaling †	3.05	*BMPR2, CSNK2A1, IL1A, IL1R2, IL18, IRAK4, PIK3C2A, RIPK1, TGFA, TGFBR1, TLR4, TNFRSF1A, TNFSF13B*
Th1 and Th2 Activation Pathway †	-	*BMPR2, CCR1, CD247, CD274, IFNGR1, IL4R, IL6R, IL18, JAK2, NOTCH2, PIK3C2A, SOCS3, TGFBR1*
T Helper Cell Differentiation †	-	*BCL6, IFNGR1, IL4R, IL6R, IL18, TGFBR1, TNFRSF1A*
Th1 Pathway *	1.41	*CD247, CD274, IFNGR1, IL6R, IL18, JAK2, NOTCH2, PIK3C2A, SOCS3*
Fcγ Receptor mediated Phagocytosis in Macrophages and Monocytes †	2.12	*ARPC2, FGR, GAB2, HCK, HMOX1, NCF1, PTEN, PTK2B*
Granulocyte Adhesion and Diapedesis †	-	*CCL14, CSF3R, CXCR2, IL1A, IL1R2, IL1RAP, IL18, IL18RAP, ITGAM, SELL, TNFRSF1A*
Role of Pattern Recognition Receptors in Recognition of Bacteria and Viruses *	2.00	*C3AR1, DDX58, IL1A, IL18, NOD1, OAS1, PIK3C2A, TLR4*
LXR/RXR Activation ‡	2.53	*ABCA1, ARG2, FDFT1, IL1A, IL1R2, IL1RAP, IL18, IL18RAP, LY96, MYLIP, TLR4, TNFRSF1A*
LPS/IL-1 Mediated Inhibition of RXR Function †	1.89	*ABCA1, ACSL1, ACSL4, ALDH1L2, GSTM3, IL1A, IL1R2, IL1RAP, IL18, IL18RAP, LY96, RARA, TLR4, TNFRSF1A*
PPAR Signaling *	2.65	*IL1A, IL1R2, IL1RAP, IL18, IL18RAP, SOS2, TNFRSF1A*
**Signaling**		
RhoA Signaling *	0.38	*ABL2, ARHGAP6, ARPC2, LIMK2, LPAR6, PIP5K1B, PPP1R12A, PTK2B*
p38 MAPK Signaling †	2.83	*IL1A, IL1R2, IL1RAP, IL18, IL18RAP, IRAK4, RPS6KA3, TGFBR1, TNFRSF1A*
TREM1 Signaling *	2.45	*CASP5, IL18, JAK2, NOD1, TLR4, TREM1*
iNOS Signaling *	2.24	*IFNGR1, IRAK4, JAK2, LY96, TLR4*

The significance of association between the data set and pathway: * *p* < 0.05; † *p* < 0.01; ‡ *p* < 0.001. Positive and negative *z*-scores suggest activation or inhibition, respectively. Missing *z*-scores have not been calculated due to lack of database information. Gene symbols in bold font: H > L; gene symbols in normal font: H < L. EIF—Eukaryotic initiation factor, CDP—Cytidine diphosphate, IL—Interleukin, NF-κB—Nuclear factor κB, Th—T helper cell, LXR—Liver X receptor, RXR—Retinoid X receptor, LPS—Lipopolysaccharide, PPAR—Peroxisome proliferator-activated receptors, RhoA—Ras homolog gene family, member A, MAPK—Mitogen-activated protein kinase, TREM1—Triggering receptor expressed on myeloid cells 1, iNOS—Inducible nitric oxide synthase.

**Table 3:** Transcripts showing the highest differences in mRNA abundance between animals fed the L and H diets.

Gene Symbol	FC ^1^	*p* Value	*q* Value	Function
*IL22RA2*	5.95	<0.001	0.075	cytokine receptor
*RNF128*	3.55	<0.001	0.102	transmembrane protein/ligase
*ARG2*	3.40	<0.001	0.087	arginine metabolism
*C3AR1*	3.22	0.001	0.123	G-protein coupled receptor
*IL1R2*	3.14	0.002	0.144	interleukin receptor
*SPTSSA*	−3.81	<0.001	0.102	sphingolipid biosynthesis
*PLPP3*	−2.09	0.003	0.151	membrane glycoprotein
*EEF1A1*	−1.84	0.007	0.186	elongation factor
*PEBP1*	−1.81	0.009	0.201	binding protein
*APEX1*	−1.74	0.003	0.151	endonuclease

^1^ Fold change; positive FC (L < H); negative FC (L > H); IL22RA2—Interleukin 22 receptor subunit alpha 2; RNF128—ring finger protein 128, E3 ubiquitin protein ligase; ARG2—arginase 2; C3AR1—complement C3a receptor 1; IL1R2—interleukin 1 receptor type 2; SPTSSA—serine palmitoyltransferase small subunit A; PLPP3—phospholipid phosphatase 3; EEF1A1—eukaryotic translation elongation factor 1 alpha 1; PEBP1—phosphatidylethanolamine binding protein 1; APEX1—apurinic/apyrimidinic endodeoxyribonuclease 1.

## Supplementary Materials

The following are available online at http://www.mdpi.com/2072-6643/11/2/436/s1, Table S1: Analyzed nutrient composition of the experimental diets. Table S2: Performance and feed intake of pigs fed variable dietary amounts of calcium and phosphorus. Table S3: P, Ca and Zn intake of pigs fed variable dietary amounts of calcium and phosphorus throughout the feeding trial. Table S4: Full list of pathways in PBMC of pigs fed diets with low and high amounts of calcium and phosphorus affected by differentially expressed genes.
