# Supplementary material for: Physiological and Transcriptional Responses in Weaned Piglets Fed Diets with Varying Phosphorus and Calcium Levels
Source: Nutrients. 2019 Feb 20;11(2):436. doi: 10.3390/nu11020436 (PMC6412343; doi:10.3390/nu11020436)
Supplement: Supplementary file 1 [file nutrients-11-00436-s001.zip › nutrients-442268-supplementary/Table S1 .pdf]

Table S1 Analyzed nutrient composition of the experimental diets.

Component	L	M	H
Dry matter (DM) (g/kg FM)	898	897	905
Crude ash (g/kg DM)	49.7	61.5	79.0
Crude protein (g/kg DM)	196.0	196.2	209.9
Crude fiber (g/kg DM)	52.0	48.9	49.7
Crude fat (g/kg DM)	35.6	33.7	31.7
Sugar (g/kg DM)	67.6	71.0	69.8
Starch (g/kg DM)	393.1	399.1	383.4
Energy (MJ/kg DM)	14.7	14.6	14.3
Calcium (g/kg DM)	7.9	12.7	16.9
Phosphorus (g/kg DM)	5.7	8.4	10.2
Phosphorus sol. (g/kg DM)	3.1	4.9	6.0
Ca:P Ratio	1.6	1.7	1.8
Zinc (mg/kg DM)	36.9	37.0	39.1

FM – Fresh matter; DM – Dry matter; L – Low P diet; M – Medium P diet; H – High P diet
