# Supplementary material for: Physiological and Transcriptional Responses in Weaned Piglets Fed Diets with Varying Phosphorus and Calcium Levels
Source: Nutrients. 2019 Feb 20;11(2):436. doi: 10.3390/nu11020436 (PMC6412343; doi:10.3390/nu11020436)
Supplement: Supplementary file 1 [file nutrients-11-00436-s001.zip › nutrients-442268-supplementary/Table S2 .pdf]

Table S2 Performance and feed intake of pigs fed variable dietary amounts of calcium and phosphorus. Data refer to the weekly documentation of body weight, body weight gain and feed intake throughout the feeding trial.

**Body weight (kg)**							
**Age**	**L**		**M**		**H**		**p-Value**
	**Mean**	**SD**	**Mean**	**SD**	**Mean**	**SD**	
28 dpn	8.49	1.67	8.42	1.50	8.42	0.81	0.991
35 dpn	9.61	1.57	9.43	1.36	9.57	0.94	0.949
42 dpn	11.74	1.90	11.65	1.78	11.63	1.08	0.983
49 dpn	14.71	2.29	14.30	2.32	13.60	1.48	0.441
56 dpn	18.49[a]	2.28	17.59[a]	3.36	14.65[b]	2.04	0.020
63 dpn	22.00[a]	2.51	20.58[a]	4.26	14.95[b]	3.01	0.002
**Daily body weight gain (g/day)**							
**Trial week**	**L**		**M**		**H**		**p-Value**
	**Mean**	**SD**	**Mean**	**SD**	**Mean**	**SD**	
28-35 dpn	160.29	42.05	144.43	73.02	164.29	52.93	0.7788
35-42 dpn	304.00	61.34	316.71	64.70	294.29	51.54	0.8042
42-49 dpn	448.00[a]	95.45	378.29[a]	107.61	280.43[b]	95.95	0.0081
49-56 dpn	515.29[a]	36.11	470.00[a]	200.54	151.00[b]	129.61	0.0005
56-63 dpn	585.57[a]	90.65	499.14[a]	276.19	49.43[b]	179.78	0.0003
**Daily feed intake (g/day)**							
**Trial week**	**L**		**M**		**H**		**p-Value**
	**Mean**	**SD**	**Mean**	**SD**	**Mean**	**SD**	
28-35 dpn	224.43	56.73	196.29	69.92	197.14	44.69	0.568
35-42 dpn	482.43	96.35	443.43	69.30	486.29	95.90	0.490
42-49 dpn	786.86[a]	148.31	682.00[a,b]	120.68	671.71[b]	96.56	0.078
49-56 dpn	950.71[a]	92.89	869.14[a]	200.63	724.14[b]	94.13	0.007
56-63 dpn	1048.86[a]	166.85	958.14[a]	136.02	668.86[b]	135.57	0.001

[a,b] Indicate significant differences between groups ($p < 0.05$); L – Low P diet; M – Medium P diet; H – High P diet. dpn=days post natum
