# Supplementary material for: Physiological and Transcriptional Responses in Weaned Piglets Fed Diets with Varying Phosphorus and Calcium Levels
Source: Nutrients. 2019 Feb 20;11(2):436. doi: 10.3390/nu11020436 (PMC6412343; doi:10.3390/nu11020436)
Supplement: Supplementary file 1 [file nutrients-11-00436-s001.zip › nutrients-442268-supplementary/Table S3 .pdf]

Table S3 P, Ca and Zn intake of pigs fed variable dietary amounts of calcium and phosphorus throughout the feeding trial.

Calcium intake (g/day)

Trial week	L		M		H	
	Mean	SD	Mean	SD	Mean	SD
28-35 dpn	1.59[a]	0.40	2.24[a]	0.80	3.02[b]	0.68
35-42 dpn	3.42[a]	0.68	5.05[b]	0.79	7.44[c]	1.47
42-49 dpn	5.58[a]	1.05	7.77[b]	1.37	10.27[c]	1.48
49-56 dpn	6.74[a]	0.66	9.90[b]	2.29	11.08[b]	1.44
56-63 dpn	7.44[a]	1.18	10.92[b]	1.55	10.23[b]	2.07

Calcium intake (g/day/kgBW)

Trial week	L		M		H	
	Mean	SD	Mean	SD	Mean	SD
28-35 dpn	0.17[a]	0.07	0.24[a]	0.10	0.31[b]	0.06
35-42 dpn	0.29[a]	0.03	0.44[b]	0.05	0.64[c]	0.10
42-49 dpn	0.38[a]	0.04	0.54[b]	0.05	0.75[c]	0.04
49-56 dpn	0.37[a]	0.02	0.56[b]	0.09	0.77[c]	0.14
56-63 dpn	0.34[a]	0.03	0.54[b]	0.10	0.70[c]	0.19

Phosphorus intake (g/day)

Trial week	L		M		H	
	Mean	SD	Mean	SD	Mean	SD
28-35 dpn	1.15[a]	0.29	1.48[a,b]	0.53	1.82[b]	0.41
35-42 dpn	2.47[a]	0.49	3.34[b]	0.52	4.49[c]	0.89
42-49 dpn	4.03[a]	0.76	5.14[b]	0.91	6.20[c]	0.89
49-56 dpn	4.87[a]	0.48	6.55[b]	1.51	6.68[b]	0.87
56-63 dpn	5.37[a]	0.85	7.22[b]	1.02	6.17[a,b]	1.25

Phosphorus intake (g/day/kgBW)

Trial week	L		M		H	
	Mean	SD	Mean	SD	Mean	SD
28-35 dpn	0.12[a]	0.05	0.16[a,b]	0.06	0.19[b]	0.04
35-42 dpn	0.21[a]	0.02	0.29[b]	0.03	0.39[c]	0.06
42-49 dpn	0.27[a]	0.03	0.36[b]	0.04	0.46[c]	0.02
49-56 dpn	0.26[a]	0.02	0.37[b]	0.06	0.46[c]	0.09
56-63 dpn	0.24[a]	0.02	0.36[b]	0.06	0.43[b]	0.12

Zinc intake (g/day)

Trial week	L		M		H	
	Mean	SD	Mean	SD	Mean	SD
28-35 dpn	7.44	1.88	6.51	2.32	6.98	1.58
35-42 dpn	15.99	3.19	14.72	2.30	17.21	3.39
42-49 dpn	26.07	4.91	22.63	4.01	23.77	3.42
49-56 dpn	31.50	3.08	28.85	6.66	25.62	3.33
56-63 dpn	34.76[a]	5.53	31.80[a]	4.51	23.67[b]	4.80

Zinc intake (g/day/kgBW)

Trial week	L		M		H	
	Mean	SD	Mean	SD	Mean	SD

28-35 dpn	0.80	0.31	0.70	0.28	0.73	0.14
35-42 dpn	1.36[a,b]	0.16	1.27[a]	0.15	1.48[b]	0.24
42-49 dpn	1.77[a]	0.19	1.59[b]	0.16	1.74[a,b]	0.09
49-56 dpn	1.71	0.11	1.64	0.26	1.77	0.33
56-63 dpn	1.58	0.15	1.58	0.28	1.63	0.45

[a,b,c] Indicate significant differences between groups ($p < 0.05$); L – Low P diet; M – Medium P diet; H – High P diet. dpn=days post natum
