# Supplementary material for: Physiological and Transcriptional Responses in Weaned Piglets Fed Diets with Varying Phosphorus and Calcium Levels
Source: Nutrients. 2019 Feb 20;11(2):436. doi: 10.3390/nu11020436 (PMC6412343; doi:10.3390/nu11020436)
Supplement: Supplementary file 1 [file nutrients-11-00436-s001.zip › nutrients-442268-supplementary/Table S4 .pdf]

Table S4 Full list of pathways in PBMC of pigs fed diets with low and high amounts of calcium and phosphorus affected by differentially expressed genes. The threshold was set at –log (B-H p-Value) of 1.31 after Benjamini-Hochberg correction.

Canonical Pathway	-log (B-H p-value)	z-score	Molecules
IL-10 Signaling	4.1		**ARG2, CCR1, HMOX1, IL1A, IL1RAP, IL1R2, IL4R, IL18, IL18RAP SOCS3**
LXR/RXR Activation	3.7	-2.53	**ARG2, ABCA1,** FDFT1, **IL1A, IL1RAP, IL1R2**, **IL18, IL18RAP, LY96, MYLIP, TLR4, TNFRSF1A**
IL-6 Signaling	3.66	2.89	CSNK2A1, **IL1A, IL1RAP, IL1R2, IL6R, IL18, IL18RAP, JAK2, PIK3C2A, SOCS3, SOS2, TNFRSF1A**
Hepatic Fibrosis / Hepatic Stellate Cell Activation	3.54		**ECE1, IFNGR1, IL1A, IL1RAP, IL1R2, IL4R, IL6R, IL18RAP, LY96, TGFA, TGFBR1, TIMP2, TLR4, TNFRSF1A**
Acute Phase Response Signaling	3.31	2.11	**C4BPA,** F8, **HMOX1, IL1A, IL1RAP, IL6R, IL18, JAK2, RIPK1, SOCS3, SOD2, SOS2, TNFRSF1A**
NF-κB Signaling	3.09	3.05	**BMPR2,** CSNK2A1, **IL1A, IL1R2, IL18, IRAK4, PIK3C2A, RIPK1, TGFA, TGFBR1, TLR4, TNFRSF1A, TNFSF13B**
LPS/IL-1 Mediated Inhibition of RXR Function	2.82	1.89	**ABCA1, ACSL1, ACSL4, ALDH1L2,** GSTM3, **IL1A, IL1RAP, IL1R2, IL18, IL18RAP, LY96, RARA, TLR4, TNFRSF1A**
CDP-diacylglycerol Biosynthesis I	2.67		**CDS1, GPAT3, GPAT4, LPCAT2, MBOAT7**
Phosphatidylglycerol Biosynthesis II (Non-plastidic)	2.55		**CDS1, GPAT3, GPAT4, LPCAT2, MBOAT7**
Th1 and Th2 Activation Pathway	2.55		**BMPR2, CCR1,** CD247, **CD274, IFNGR1, IL4R, IL6R, IL18, JAK2, NOTCH2, PIK3C2A, SOCS3, TGFBR1**
Role of Macrophages, Fibroblasts and Endothelial Cells in Rheumatoid Arthritis	2.55		**IL1A, IL1RAP, IL1R2, IL6R, IL17RA, IL18, IL18RAP, IRAK4, JAK2, LRP1, PIK3C2A, RIPK1, SOCS3, TLR4, TNFRSF1A, TNFSF13B**
Triacylglycerol Biosynthesis	2.42		**DGAT2, GPAT3, GPAT4, LPCAT2,** PLPP3, **MBOAT7**
p38 MAPK Signaling	2.3	2.82	**IL1A, IL1RAP, IL1R2, IL18, IL18RAP, IRAK4, RPS6KA3, TGFBR1, TNFRSF1A**
Fcγ Receptor-mediated Phagocytosis in Macrophages and Monocytes	2.3	2.12	ARPC2, **FGR, GAB2, HCK, HMOX1, NCF1, PTEN, PTK2B**
Granulocyte Adhesion and Diapedesis	2.25		**CCL14, CSF3R, CXCR2, IL1A, IL1RAP, IL1R2, IL18, IL18RAP, ITGAM, SELL, TNFRSF1A**
T Helper Cell Differentiation	2.25		**BCL6, IFNGR1, IL4R, IL6R, IL18, TGFBR1, TNFRSF1A**
Hepatic Cholestasis	2.08		**IL1A, IL1RAP, IL1R2, IL18, LY96, RARA, TLR4, TNFRSF1A, IL18RAP, IRAK4**
EIF2 Signaling	2.08	-1.13	**ATF3,** EIF3K, **PIK3C2A,** RPL7, RPL12, RPL24, RPL34, RPSA, RPS8, RPS19, RPS26, **SOS2**
D-myo-inositol	1.86		ACP1, **DUSP2, IPMK, PPP1R12A, PTEN, PTPN12,**

(1,4,5,6)-Tetrakisphosphate Biosynthesis			SET, **SOCS3, TNS3**
D-myo-inositol (3,4,5,6)-tetrakisphosphate Biosynthesis	1.86		ACP1, **DUSP2, IPMK, PPP1R12A, PTEN, PTPN12,** SET, **SOCS3, TNS3,**
iNOS Signaling	1.82	2.24	**IFNGR1, IRAK4, JAK2, LY96, TLR4**
PPAR Signaling	1.77	-2.65	**IL1A, IL1RAP, IL1R2, IL18, IL18RAP, SOS2, TNFRSF1A,**
RhoA Signaling	1.76	0.38	ABL2, **ARHGAP6,** ARPC2, **LIMK2, LPAR6, PIP5K1B, PPP1R12A, PTK2B**
Role of Osteoblasts, Osteoclasts and Chondrocytes in Rheumatoid Arthritis	1.53		**ADAM17, BMPR2, IL1A, IL1RAP, IL1R2, IL18, IL18RAP, LRP1, PIK3C2A, PTK2B, TNFRSF1A**
Th1 Pathway	1.53	1.13	CD247**, CD274, IFNGR1, IL6R, IL18, JAK2, NOTCH2, PIK3C2A, SOCS3**
3-phosphoinositide Biosynthesis	1.53		ACP1, **DUSP2, PIK3C2A, PIP5K1B, PPP1R12A, PTEN, PTPN12,** SET, **SOCS3, TNS3**
Superpathway of Inositol Phosphate Compounds	1.53		ACP1, **DUSP2, IPMK, PIK3C2A, PIP5K1B, PPP1R12A, PTEN, PTPN12,** SET, **SOCS3, TNS3**
Role of Pattern Recognition Receptors in Recognition of Bacteria and Viruses	1.52	2.00	**C3AR1, DDX58, IL1A, IL18, NOD1, OAS1, PIK3C2A, TLR4**
Type I Diabetes Mellitus Signaling	1.47	1.89	CD247, **IFNGR1, IL1RAP, JAK2, RIPK1, SOCS3, TNFRSF1A**
Interferon Signaling	1.44	2.00	**IFIT3, IFNGR1, JAK2, OAS1**
MSP-RON Signaling Pathway	1.44		**CSF2RB, ITGAM, JAK2, PIK3C2A, TLR4**
Th2 Pathway	1.35	0.70	**BMPR2, CCR1,** CD247, **IL4R, JAK2, NOTCH2, PIK3C2A, SOCS3, TGFBR1**
PDGF Signaling	1.34	0.82	ABL2, ACP1, CSNK2A1, **JAK2, PIK3C2A, SOS2**

The significance of association between the data set and pathway: *p < 0.05; †p < 0.01; ‡p < 0.001. Positive and negative z-scores suggest activation or inhibition, respectively. Missing z-scores have not been calculated due to lack of database information. Gene symbols in bold font: H > L; gene symbols in normal font: H < L.
